# Cholesterol Remnants, Triglyceride-Rich Lipoproteins and Cardiovascular Risk

**DOI:** 10.3390/ijms24054268

**Published:** 2023-02-21

**Authors:** Francesco Baratta, Nicholas Cocomello, Mattia Coronati, Domenico Ferro, Daniele Pastori, Francesco Angelico, Maria Del Ben

**Affiliations:** Department of Clinical Internal, Anaesthesiological and Cardiovascular Sciences, Policlinico Umberto 1, Sapienza University, 00161 Rome, Italy

**Keywords:** remnant cholesterol, triglyceride-rich lipoprotein, residual cardiovascular risk, atherosclerosis

## Abstract

Randomized clinical trials with statins and other lipid-lowering drugs have shown the presence of a “residual cardiovascular risk” in those treated to “target” for LDL-cholesterol. This risk is mainly associated to lipid components other than LDL and in particular to remnant cholesterol (RC) and to lipoproteins rich in triglycerides in fasting and non-fasting conditions. During fasting, RCs correspond to the cholesterol content of the VLDL and their partially depleted triglyceride remnant containing apoB-100. Conversely, in non-fasting conditions, RCs include also cholesterol present in chylomicrons containing apoB-48. Therefore, RCs refer to total plasma cholesterol minus HDL-cholesterol and LDL-cholesterol, that is, all the cholesterol present in the VLDL, chylomicrons and in their remnants. A large body of experimental and clinical data suggests a major role of RCs in the development of atherosclerosis. In fact, RCs easily pass the arterial wall and bind to the connective matrix stimulating the progression of smooth muscle cells and the proliferation of resident macrophages. RCs are a causal risk factor for cardiovascular events. Fasting and non-fasting RCs are equivalent for predicting vascular events. Further studies on drugs effect on RC levels and clinical trials to evaluate the efficacy of RC reduction on cardiovascular events are needed.

## 1. Introduction

Over the last few years, the importance of controlling the “residual risk”, in subjects who have reached the LDL cholesterol (LDL-C) “target”, during cholesterol-lowering therapy, has been emphasized [1]. In fact, the results of statin trials have demonstrated that even after maximal treatment with statins and/or lipid-lowering combination therapy, a significant residual cardiovascular risk often remains [2]. While in the past the residual risk was considered as the consequence of the still elevated LDL-C serum levels after statin treatment, recently, the concept of residual risk has been extended to the risk associated with known and modifiable but not completely corrected and correctable lipid and non-lipid risk factors (e.g., HDL-C and triglycerides (TG) levels, lipoprotein (a), abdominal adiposity, blood pressure, insulin resistance, smoking, fatty liver etc.) [3,4]. More recently, the concept of residual risk has been extended also to the risk associated with factors not yet fully elucidated (e.g., inflammation, oxidative stress, liver fibrosis, etc.).

Multiple lines of evidence suggest that triglyceride-rich lipoproteins (TGRLs) play a central role in residual cardiovascular risk. In particular, it has been suggested that remnant cholesterol (RC) levels are likely contributors to the residual risk in patients with very well controlled LDL-C levels on intensive lipid lowering therapy. RCs are probably the most important determinants of cardiovascular risk after LDL-C levels. In fact, plasma concentration of remnants appears to be related to cardiovascular risk independently of LDL-C.

Moreover, both LDL-TG and RCs, but not LDL-C are related to systemic low-grade inflammation and vascular damage [5,6] and it has been proposed that the TG content of LDL may be a marker of delayed remnant particle catabolism. Consistent with these observations, individuals with increased LDL-TG and RCs concentrations have also increased concentrations of inflammatory marker hs-CRP and white blood cell count [7], which may reflect an adverse impact of inflammation. Additionally, beyond residual cholesterol risk, the results of clinical trials also support the concept of residual inflammation risk. In fact, in both Prove It [8] and Improve IT [9] trials almost one-third of statin-treated patients had hs-CRP > 2 mg/L. Therefore, accumulating evidences from epidemiological and genetic studies, as well as randomized clinical trials, suggest that remnant lipoproteins and inflammation, as well as LP (a), are causally related to risk of ACVD in individuals already treated with statin therapy [10].

So far, many papers analysed oxidative stress as the critical link between lipids, inflammation and the pathogenesis of atherosclerosis. In fact, minimizing oxidant stress would significantly reduce the trapping of Apolipoprotein B in the intima, mitigate the association between lipids and chronic inflammation and impair the pathogenesis of atherosclerosis [11].

More recently, also liver fibrosis has been proposed as a new player in the evaluation of residual cardiovascular risk [12]. In fact, the hypothesis has been suggested that hepatic fibrosis may be the result of long-term exposure to cardio-metabolic risk factors such as obesity, dyslipidaemia, diabetes, and metabolic syndrome [13]. These conditions can induce insulin resistance, low-grade inflammation, and oxidative stress which in turn can induce hepatocellular damage, activation of stellate cells and Kupfer cells with consequent fibrogenesis. Therefore, liver fibrosis could be interpreted as a non-lipid marker of residual cardiovascular risk.

## 2. Triglyceride Rich Lipoproteins and Cholesterol Remnant

TGRLs are macromolecules composed of a large neutral lipid (TG) core surrounded by polar components including phospholipids, free cholesterol, and apolipoproteins. TGRLs originate from the intestine (chylomicrons) and the liver (very-low-density lipoproteins—VLDL) and are the main source of fatty acids for energy production in peripheral tissues or for storage in adipose tissue. TGRLs transport mainly TG and may significantly contribute to residual cardiovascular risk in patients at target for LDL-C. Approximately 20% of the general population has elevated serum TG and there is a wealth of evidence to indicate that TG are an independent risk factor for cardiovascular events [14,15,16].

Serum TG strongly correlated with TGRLs concentration both in fasting and non-fasting conditions. However, it has been suggested that the cardiovascular risk associated with TGRLs is mainly to be referred to their content in RC. RC are probably the biggest determinants of cardiovascular risk after LDL-C levels. In fact, the plasma concentration of remnants appears to be related to cardiovascular risk independently of LDL-C. Apolipoprotein B100 is the major structural protein in TGRLs secreted by the liver, while apolipoprotein B-48 is the main apoprotein in chylomicrons secreted in the intestine.

**Figure 1:**
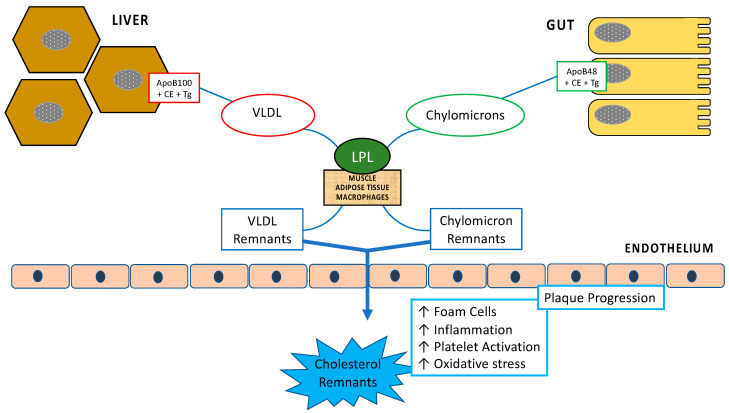
Remnants of cholesterol and atherosclerosis: During the post-prandial period, enterocytes produce chylomicrons which are released into the circulation via the intestinal lymphatic system. In the liver, regardless of meals, hepatocytes synthesize and release VLDL into the general circulation. Circulating and tissue lipoprotein lipases (LPL) hydrolyse VLDL and chylomicron triglycerides, transforming them into VLDL/IDL remnants and chylomicron remnants, respectively. Due to their small size, remnants easily penetrate the arterial wall and accumulate in the sub-endothelium causing inflammation, foam cells formation, platelet activation and oxidative stress leading to plaque progression.

TGRLs and their remnants accumulate in the plasma when plasma TG is severely elevated. Increased liver TG secretion is the main determinant in moderate hypertriglyceridemia, whereas reduced lipoprotein-mediated TG lipolysis is the dominant abnormality in the presence of severe hypertriglyceridemia. During lipolysis, the TGRLs core content of TG decreases and cholesterol esters increase due to cholesteryl ester transfer protein (CETP)-mediated exchange of TGRL-TG for LDL- and high-density lipoprotein (HDL)-cholesterol esters. Therefore, remnant lipoproteins are enriched in both free and esterified cholesterol. Remnant-like lipoproteins are always present in the blood stream, both in fasting and non-fasting conditions. They constitute a heterogeneous group of lipoproteins with variable size and composition (Figure 1).

The amount of TGRL derived remnants may be indirectly assessed by measuring their cholesterol content. In fasting conditions, RC corresponds to the cholesterol content present in VLDL synthesized in the liver and in their partially depleted triglyceride remnants (IDL), containing apoB-100. Conversely, in non-fasting conditions, RC also includes, in addition to lipoproteins defined above, cholesterol present in the chylomicrons resulting from the intestinal absorption of dietary fats, containing apoB-48. In summary, we can consider as RC all plasma cholesterol minus HDL-C and LDL-C, that is, all cholesterol present in VLDL, chylomicrons and their remnants, globally defined as TGRLs. RCs represent one-third of the total serum cholesterol concentration. After a fatty meal, lipolysis of intestinal chylomicrons induces the production of remnants which increase significantly and proportionally with the level of fasting TG. Circulating and tissue lipoproteinlipases (LPLs) hydrolyse VLDL and chylomicron TG, transforming them into VLDL/IDL remnants and chylomicron remnants, respectively, which are relatively cholesterol-enriched. Therefore, remnant particles peak early in the post-prandial state but are rapidly cleared from plasma when metabolism is intact. In clinical conditions characterized by hypertriglyceridemia, such as obesity, diabetes, metabolic syndrome and non-alcoholic fatty liver disease, both chylomicrons and VLDL overproduction and inefficient lipolysis lead to increased lipoprotein remnant generation. According to the European joint consensus statement from the European Atherosclerosis Society (EAS), fasting RC should be <0.8 mmol/L in fasting condition and not exceed 0.9 mmol/L [17] in the postprandial status. RC, together with lipoprotein (a) and markers of inflammation are causally associated with increased residual cardiovascular risk in statin-treated patients at target for LDL-C [10].

## 3. The Measurement of Non-Fasting Lipids

Traditionally, plasma lipids have always been evaluated in the fasting state and only fasting lipid profiles have been used to evaluate cardiovascular risk and to characterize primary and secondary dyslipidaemias. Moreover, only fasting LDL-C goals have been indicated by Guidelines as therapeutic targets. However, over the last few years, several authors have recommended measuring lipids in non-fasting conditions and most recent AHA/ACC guidelines recommends using either fasting or non-fasting blood to assess lipid profile [18,19,20,21,22,23].

In the Copenhagen General Population Study, lipid profiles changed minimally in response to food intake and non-fasting lipid profiles predicted increased risk of cardiovascular events (CVD) [24]. In a prospective study of 26,330 healthy women (19,983 fasting; 6347 non-fasting), associations of baseline lipids with incident CVD (754 fasting; 207 non-fasting) were examined over an 11-year follow-up [25]. HDL C, TG, total/HDL-C ratio, and apolipoprotein A-1 predicted CVD when measured non-fasting. By contrast, total, LDL, and non-HDL-C, in addition to apolipoprotein B-100 and B-100/A-1 ratio, provided less useful CVD risk information when non-fasting. Moreover, in 8270 participants in ASCOT-LLA, in the same individuals, non-fasting lipids were better than or equivalent to fasting ones for predicting incident CVD [19].

Recently, the European Atherosclerosis Society and the European Federation of Clinical Chemistry and Laboratory Medicine concluded that the measurement of plasma lipids in non-fasting conditions is at least valid as those measured at fasting for cardiovascular risk assessment [22]. It was also emphasized that fasting is not always necessary for a correct evaluation of lipids. To date, non-fasting lipid assessment is only recommended in Denmark, where fasting is not required to perform a standard lipid profile and where clinicians are offered the option of lipid retesting if TG values exceed 350 mg/dL [22]. This offers clear advantages to doctors and patients, especially from a practical point of view. On the other hand, it should be considered that most people spend most of the day in a non-fasting condition. Finally, fasting may mask TGRLs abnormalities in some patients, such as diabetics.

## 4. How to Measure Cholesterol Remnants

Accurate measurement of RCs is difficult due to the heterogeneity of lipoprotein composition, size and density, as well as to their rapid metabolization. RC measurement by separating lipoproteins with ultracentrifugation or polycrylamide gel electrophoresis is complex and is rarely performed in the clinical setting. Conceptually, RCs represent the non-HDL and non-LDL circulating cholesterol. In both clinical practice and epidemiology, and only in fasting conditions, it has been proposed to roughly calculate plasma RCs levels by subtracting HDL-C and LDL-C values from total cholesterol. Excluding severe hypertriglyceridemia, where Frieldwald formula [26] is inapplicable, RCs values are easily computable starting from routine laboratory values [26]. In absence of dosed LDL-C, considering the 1 to 5 ratio between cholesterol and triglyceride content in VLDL under fasting conditions, we can easily calculate RCs dividing serum triglycerides by 5. Recently, a new method for calculating fasting LDL-C has been proposed, which allows a more accurate estimate of the values than those derived from the Friedwald formula [26]. This method is useful in conditions of serum low LDL-C and high TG which are observed with increasing frequency because of the increasingly effectiveness of LDL-lowering therapies and the growing prevalence of obesity and metabolic syndrome (19). This new calculation method suggests applying a correction factor to VLDL C/TG ratio, which in the Friedwald formula is calculated as 1:5, based on the individual values of serum non-HDL C and TG [27].

In addition to calculation methods, RC can be directly measured using a variety of analytical methods including ultracentrifugation, nuclear magnetic resonance (NMR) spectroscopy, and by a direct automated assay. A quantitative method for assessing the concentration of RC in fasting conditions is based on the immunoseparation of remnant-like particles in serum using monoclonal antibodies to apo B-100 and apoA1 [28]. The measurement of RC derived from the exogenous pathway (i.e., chylomicron remnants) can be performed via ultracentrifugation or, indirectly, by measuring apoB-48. Recently, within the Copenhagen General Population Study, it has been demonstrated that directly measured RC identifies more accurately patients at higher risk of myocardial infarction, in comparison to calculated ones [29].

In non-fasting conditions, RC evaluation is more complex since remnants deriving from intestinal chylomicrons are added to VLDL remnants. In this condition, RCs are strongly correlated with TG serum levels which vary according to lipid meals composition and to fasting length since the last meal. In fact, after a fatty meal, apo-B48 levels, TGRLs-C and TG rapidly increase, while there is no postprandial increase in the apoB-100 and LDL-C values. For this reason, postprandial apoB-48 values could be used as a very sensitive marker of non-fasting triglyceridemia, using immunoturbidimetric and immunonephelometric assays commercially available on commonly used automated systems from several manufacturers [30].

RC serum levels vary little between fasting and postprandial periods. Therefore, even in non-fasting condition, RC levels can be accurately calculated by subtracting HDL-C and LDL-C from total cholesterol. To date, RCs in most studies have only been evaluated in fasting conditions and we have little information regarding RC and LRT concentration during the postprandial period. Both increase after a fatty meal, mostly in subjects with primary or secondary lipoprotein metabolism alterations and in those with insulin resistance. However, at the present, we do not yet have a standardized oral fatty meal test for the evaluation of lipid changes during the postprandial phase.

Finally, there is a strong urgency for the development of automated direct analysis methods given the importance of remnants evaluation in the definition of residual cardiovascular risk [30].

## 5. Cholesterol Remnants and Their Role in Atherogenesis

Remnant lipoproteins are cleared by the liver mainly via LDL receptor or penetrate arterial wall, thus contributing to the initiation and progression of atherosclerotic lesions. Unlike chylomicrons and VLDLs which do not penetrate the vessel wall due to their large size, and similarly to LDLs, LRTs remnants, due to the small size, easily pass through the arterial wall and are retained in the sub-endothelium, where they bind to the connective matrix and promote inflammation. Their presence stimulates the progression of smooth muscle cells and the proliferation of resident macrophages [31,32]. However, unlike LDLs, TGRLs are directly degraded by scavenger receptors in macrophages in the absence of oxidative modifications thus contributing to plaque formation and progression. RCs can also induce endothelial dysfunction, the production of adhesion molecules and platelet activation, as well as increase the expression of CD40 and metalloproteins [33,34]. Finally, high levels of non-fasting RCs can increase the expression of inflammatory interleukins and cytokines, increase the inflammatory response of monocytes and induce the appearance of a chronic low-grade inflammation, which, on the contrary, is not observed in the presence of high levels of LDL-C [6,35].

## 6. Cholesterol Remnants and Cardiovascular Events

Several studies examined the atherogenic role of RCs (Table 1). Since 2001, a positive association was described, independent of traditional risk factors, between TGRLs-remnant levels and the ischemic heart disease prevalence in a sample of women from the Framingham Heart Study [36]. Subsequently, Fukuscima et al. [37] demonstrated that elevated TGRLs-C levels were a significant and independent risk factor for ischemic heart disease and type 2 diabetes mellitus.

However, it is in Denmark that the largest studies on the predictive role of TGRLs on cardiovascular events have been carried out. In a study conducted over 22 years on 90,000 Danish subjects, the progressive increases in non-fasting measured RC and LDL-C were similarly associated with the increased risk of ischemic heart disease and myocardial infarction; however, only RC levels were associated with all-cause mortality [50,51]. It was estimated that a 1 mmol/L (39 mg/dL) increase in RC in non-fasting conditions was shown to correspond to a 2.8-fold increased risk for coronary heart disease [50,51]. A subsequent study, also conducted in Denmark on a court of 5414 patients with ischemic heart disease, confirmed the association between calculated and/or measured non-fasting RC levels and all-cause mortality, while a similar association was not observed for LDL-C levels [4]. More recently, lowering RCs of 32 mg/dL and 81 mg/dL was estimated to reduce recurrent MACE by 20% and 50% respectively [52]. Finally, very recently, in a follow up study of 19,650 adults in the United States from the National Health and Nutrition Examination Survey (NHANES) (1999–2014), elevated RC levels were independently associated with cardiovascular mortality [53].

Similarly, in a series of patients with coronary heart disease treated with lipid-lowering therapies and reaching LDL-C values < 100 mg/dL, TGRL-C values predicted coronary events recurrence, suggesting RCs as a possible new target of “residual risk” [38]. Similar results were reported by Kugiyana C. et al. in 153 patients with coronary heart disease [40]. Moreover, in a TNT trial post-hoc analysis, fasting TGRLs-C values predicted cardiovascular events and 1 SD RC reduction by atorvastatin reduced cardiovascular events incidence similarly to that obtained with 1 SD LDL-C reduction [54].

The association between RCs and cardiovascular disease was confirmed in different ethnicities). In a study conducted in Japan, confirming the predictive role of CR levels on cardiovascular events recurrence in secondary prevention, RC values were used to increase the prognostic value of traditional risk factors, allowing the authors to better stratify patients’ cardiovascular risk [41]. In addition, no differences were observed in the predictive role between USA black (Jackson Heart Study) and white (Framingham Offspring Cohort Study) subjects [42]. In a Chinese community-based population, increased RC levels better associated with new-onset carotid plaque diagnosis in comparison to other conventional lipid parameters [47]. Finally, in ASCVD-free individuals from three landmark US cohorts—the Atherosclerosis Risk in Communities (ARIC) study, the Multi-Ethnic Study of Atherosclerosis (MESA), and the Coronary Artery Risk Development in Young Adults (CARDIA)—levels of RC were associated with ASCVD independently from traditional cardiovascular risk factors, such as LDL-C, and non-HDL-C or apo-B levels [45].

The good RCs performance for the stratification of cardiovascular risk in dysmetabolic patients was confirmed by several studies. The predictive role of RCs on CV events was confirmed in subjects with type 2 diabetes and chronic kidney disease [43]. Moreover, in a prospective observational cohort study including 798 unselected NAFLD patients with cardio-metabolic diseases, high RC levels were predictive of future CVD [46]. Similarly, in the PREDIMED study, in overweight or obese subjects at high cardiovascular risk, TG and RC serum levels, but not LDL-C, were associated with cardiovascular outcomes [44]. Recently, a retrospective registry of real-world patients treated with PCSK9 inhibitors demonstrated a positive effect on RCs and lipid residual risk beyond LDL-C reductions [55].

Recently, data from Copenhagen General Population Study suggested a stronger association of elevated RCs with peripheral artery disease, than that observed with coronary heart disease and ischemic stroke [48]. Data from the same population also showed that high RCs and triglyceridemia associate with cardiovascular and other non-neoplastic causes of death [49].

Interestingly, mixed results were reported using lowering triglycerides (and cholesterol remnants) drugs to reduce cardiovascular events. While the use of pemafibrate was not associated with cardiovascular benefits [56], icosapent ethyl, was found to be effective in reducing both triglycerides and cardiovascular disease incidence [57]. In addition, other large trials, which used different formulations of omega-3 fatty acids, did not report significant cardiovascular risk reduction [58]. Some authors hypotizes a putative anti-inflammatory effect of icosapent ethyl. While the ANCHOR study showed a reduction of inflammatory markers in patients with decompensated diabetes [59], data from the REDUCE-IT did not confirm this finding [60]. A wide debate on the causes of these contrasting findings is still ongoing [61].

## 7. Conclusions

Patients with high cardiovascular risk often have a lipoprotein profile characterized by the presence of high values of TGRLs and their remnants, reduced levels of HDL-C and the presence of small and dense LDLs [3,4] This lipoprotein phenotype is very frequent in subjects with metabolic syndrome and/or type 2 diabetes, in obesity and non-alcoholic fatty liver disease, that is, in conditions of insulin resistance in which it can at least partially explain the high cardiovascular risk. In particular, obesity is the main cause of TGRLs and remnant levels rise. On the basis of these observations, the most recent guidelines [62,63,64] indicate non-HDL-C as the second target for intervention after LDL-C, especially in subjects with hypertriglyceridemia, in obese subjects, in diabetics and in those with the metabolic syndrome, that is, in a large part of the general population. Non-HDL-C comprises cholesterol contained in all atherogenic lipoproteins, very low-density lipoproteins (VLDL), intermediate density lipoproteins (IDL), chylomicrons and their remnants, and apolipoprotein (a) [65].

In this context, a renewed attention was paid on TGRLs and RCs as residual cardiovascular risk factor and a possible target for cardiovascular prevention, as initially proposed by Zilversmit D.B. in the 1979 [4,66]. Indeed, measurement of cholesterol concentration in lipoprotein remnants can be easily calculated using standard lipid profile. Future trials are needed to evaluate whether therapies that target the reduction of circulating RCs levels are also effective for reducing residual cardiovascular risk.

## Figures and Tables

**Table 1:** Main epidemiological studies on cholesterol remnants (CR) and cardiovascular events.

Study	Study Population	Results
Remnant lipoprotein levels in fasting serum predict coronary events in patients with coronary artery disease Kugiyama k et al. [38]	135 patients with coronary artery disease with a maximum follow up of 36 months.	Patients with higher CR values developed a recurrence of coronary events more frequently [tertile I vs. tertile III: HR, 5.91; 95% CI, 2.0 to 17.2]
Prognostic value of remnant-like lipoprotein particle levels in patients with coronary artery disease and type II diabetes mellitus Fukushima H et al. [37]	120 patients with coronary artery disease and type 2 diabetes mellitus with a maximum follow up of 24 months.	Patients with higher CR values developed a recurrence of coronary artery disease more frequently [I quartile vs. others: HR 2.2; 95% CI, 1.2 to 6.4]
Are remnant-like particles independent predictors of coronary heart disease incidence? the Honolulu Heart Study Imke C et al. [39]	1156 Japanese–American men aged 60 to 82 followed for 17 years.	In multivariate analysis, RCs were correlated with the incidence of coronary events regardless of nonlipid risk factors and HDL-C and LDL-C levels. RCs correlated with TG.
Predictive value of remnant lipoprotein for cardiovascular events in patients with coronary artery disease after achievement of LDL-cholesterol goals Nakamura T. et al. [40]	560 patients suffering from coronary artery disease, on lipid-lowering therapy and at target for LDL-c (editor’s note: at the time of the study, target LDL-c < 100 mg/dL), followed up for an average of 33 months.	CR values were superior to non-HDL-C in predicting cardiovascular events in coronary artery patients with LDL-C < 100 mg/dL on statin therapy [As a continuous variable expressed in mg/dl: HR 1.74; 95% CI 1.31–2.32]
High remnant lipoprotein predicts recurrent cardiovascular events on statin treatment after acute coronary syndrome Nguyen S. V. et al. [41]	190 Japanese patients with coronary artery disease, and on statin treatment, followed over time for up to 70 months.	High levels of fasting CRs (5.4 mg/dL) were associated with a greater risk of recurrence of coronary events [>5.4 mg/dL: HR 2.94; 95% CI 1.40–6.18]
Remnant lipoprotein cholesterol and incident coronary heart disease: the Jackson Heart and Framingham Offspring Cohort Studies Joshi P.H. et al. [42]	4932 patients from the Framingham and Jackson Heart Studies, followed for at least 8 years.	Elevated CR levels were associated with an increased risk of ischemic cardiac events [For each SD: HR 1.23; 95% CI 1.06–1.47]
Remnant lipoproteinemia predicts cardiovascular events in patients with type 2 diabetes and chronic kidney disease Nguyen S.V. et al. [43]	365 patients with type 2 diabetes mellitus and chronic kidney disease followed for an average of 45 months	High fasting RC values (4.3 mg/dL) were associated with a higher risk of cardiovascular events [>4.3 mg/dL: HR 1.30; 95% CI 1.04–1.63]
Remnant-like particle cholesterol, low-density lipoprotein triglycerides, and incident cardiovascular disease Saeed A. et al. [7]	9334 subjects followed over time for a maximum of 16 years	CR values were associated with the incidence of cardiovascular events in univariate analysis but not after adjustment for classic risk factors.
Fasting and nonfasting lipid levels: influence of normal food intake on lipids, lipoproteins, apolipoproteins, and cardiovascular risk prediction Langsted A et al. [24]	33,391 patients from the General Copenhagen population with a follow up of 14 years	Tertile III vs tertile I of total-C., non-HDL-C, LDL-C, Apo B/TG, ratio, total-C/HDL-C Ratio, apo B/A1 predicted a 1.7 to 2.4 times increased CVD risk.
Fasting compared with nonfasting lipids and apolipoproteins for predicting incident cardiovascular events Mora S et al. [25]	26,330 healthy women followed up for 11 years in fasting and non-fasting conditions	HDL-C (HR 1.15), TG, total-C/HDL-C, Apo A 1 predicted CVD events when measured after a meal (*p* < 0.001). LDL-C, total-C and apoB100 were less predictive when measured non fasting.
Directly measured vs. calculated remnant cholesterol identifies additional overlooked individuals in the general population at higher risk of myocardial infarction Varbo A et al. [29]	16,207 patients from the General Copenhagen population with a follow up of 14 years	Measured vs. calculated RCs indicated a 1.8 (1.35–2.47) increased risk for myocardial infarction.
Remnant Cholesterol, Not LDL Cholesterol, Is Associated with Incident Cardiovascular Disease Castaner O et al. [44]	6901 high CVD risk subjects following Mediterranean diet with 14 years follow up.	In overweight/obese high CVD risk subjects TG (*p* < 0.001) and RCs, but not LDL-C were independent risk factors for CVD events.
Remnant cholesterol predicts cardiovascular disease beyond LDL and ApoB: a primary prevention study Quispe R et al. [45]	17,532 subjects with no evidence of atherosclerosis with 18.7 years follow up.	An increase in RCs was associated with the appearance of atherosclerotic lesions (HR 1.65; CI 1.45–1.89) regardless of risk factors such as LDL-C and apoB.
Remnant Lipoprotein Cholesterol and Cardiovascular and Cerebrovascular Events in Patients with Non-Alcoholic Fatty Liver Disease Pastori D et al. [46]	798 subjects with NAFLD with 32 months follow up	RCs higher in patients with NAFLD vs. controls (*p* < 0.001).
Association of remnant cholesterol and lipid parameters with new-onset carotid plaque in Chinese population Liu B at al. [47]	872 Chinese subjects with no evidence of atherosclerosis and CVD with 6.7 years follow up	RCs significantly associated with incidence of atherosclerotic plaques (OR 1.57; CI 1.03–2.45; *p* = 0.038). No association with other plasma lipids and lipoproteins.
Elevated remnant cholesterol increases the risk of peripheral artery disease, myocardial infarction, and ischaemic stroke: a cohort-based study Wadstrom B. N et al. [48]	106,937 individuals from the Copenhagen General Population Study with 15 years follow-up	Elevated RCs is associated with PAD incidence (HR 4.8; CI 3.1–7.5), more than with myocardial infarction (HR 4.2; CI 2.9–6.1) and ischaemic stroke (HR 1.8; CI 1.4–2.5)
Elevated remnant cholesterol, plasma triglycerides, and cardiovascular and non-cardiovascular mortality Wadstrom B. N et al. [49]	87,192 individuals from the Copenhagen General Population Study with 13 years follow-up	RCs of ≥1 mmol/L (39 mg/dL)band plasma triglycerides of ≥2 mmol/L (177 mg/dL) associated with two-fold mortality from cardiovascular and other non-neoplastic causes.
